# Support and Assessment for Fall Emergency Referrals (SAFER 1): Cluster Randomised Trial of Computerised Clinical Decision Support for Paramedics

**DOI:** 10.1371/journal.pone.0106436

**Published:** 2014-09-12

**Authors:** Helen Anne Snooks, Ben Carter, Jeremy Dale, Theresa Foster, Ioan Humphreys, Philippa Anne Logan, Ronan Anthony Lyons, Suzanne Margaret Mason, Ceri James Phillips, Antonio Sanchez, Mushtaq Wani, Alan Watkins, Bridget Elizabeth Wells, Richard Whitfield, Ian Trevor Russell

**Affiliations:** 1 Institute of Life Science, College of Medicine, Swansea University, Singleton Park, Swansea, United Kingdom; 2 Institute of Primary Care & Public Health, Cardiff University School of Medicine, Neuadd Meirionnydd, Heath Park, Cardiff, United Kingdom; 3 Warwick Medical School, Gibbet Hill Campus, University of Warwick, Coventry, United Kingdom; 4 East of England Ambulance Service NHS Trust, Milford Service Area, Fiveways Roundabout, Barton Mills, Suffolk, United Kingdom; 5 Swansea Centre for Health Economics, College of Human and Health Sciences, Swansea University, Singleton Park, Swansea, United Kingdom; 6 Division of Rehabilitation and Ageing, School of Community Health Sciences, University of Nottingham, Nottingham, United Kingdom; 7 School of Health and Related Research, Sheffield University, Regent Court, Sheffield, United Kingdom; 8 Department of Medicine, Cardiff University, Academic Building, Llandough Hospital, Penarth, United Kingdom; 9 Abertawe Bro Morgannwg University Health Board, Department of Stroke Medicine, Morriston Hospital, Morriston, Swansea, United Kingdom; 10 Prehospital Emergency Research Unit, Welsh Ambulance Services NHS Trust, Lansdowne Hospital, Canton, Cardiff, United Kingdom; University of Glasgow, United Kingdom

## Abstract

**Objective:**

To evaluate effectiveness, safety and cost-effectiveness of Computerised Clinical Decision Support (CCDS) for paramedics attending older people who fall.

**Design:**

Cluster trial randomised by paramedic; modelling.

**Setting:**

13 ambulance stations in two UK emergency ambulance services.

**Participants:**

42 of 409 eligible paramedics, who attended 779 older patients for a reported fall.

**Interventions:**

Intervention paramedics received CCDS on Tablet computers to guide patient care. Control paramedics provided care as usual. One service had already installed electronic data capture.

**Main Outcome Measures:**

Effectiveness: patients referred to falls service, patient reported quality of life and satisfaction, processes of care.

**Safety:**

Further emergency contacts or death within one month.

**Cost-Effectiveness:**

Costs and quality of life. We used findings from published Community Falls Prevention Trial to model cost-effectiveness.

**Results:**

17 intervention paramedics used CCDS for 54 (12.4%) of 436 participants. They referred 42 (9.6%) to falls services, compared with 17 (5.0%) of 343 participants seen by 19 control paramedics [Odds ratio (OR) 2.04, 95% CI 1.12 to 3.72]. No adverse events were related to the intervention. Non-significant differences between groups included: subsequent emergency contacts (34.6% versus 29.1%; OR 1.27, 95% CI 0.93 to 1.72); quality of life (mean SF12 differences: MCS −0.74, 95% CI −2.83 to +1.28; PCS −0.13, 95% CI −1.65 to +1.39) and non-conveyance (42.0% versus 36.7%; OR 1.13, 95% CI 0.84 to 1.52). However ambulance job cycle time was 8.9 minutes longer for intervention patients (95% CI 2.3 to 15.3). Average net cost of implementing CCDS was £208 per patient with existing electronic data capture, and £308 without. Modelling estimated cost per quality-adjusted life-year at £15,000 with existing electronic data capture; and £22,200 without.

**Conclusions:**

Intervention paramedics referred twice as many participants to falls services with no difference in safety. CCDS is potentially cost-effective, especially with existing electronic data capture.

**Trial Registration:**

ISRCTN Register ISRCTN10538608

## Introduction

Demand for immediate care through emergency ambulance services has been steadily increasing in the UK and internationally over recent years [1]. However many callers have no clinical need for treatment or investigation at an Emergency Department [2]. Although health policy in the UK encourages emergency ambulance services to offer alternatives to such callers, there is little evidence about the effectiveness, safety or cost-effectiveness of clinical assessment by paramedics and triage to other care pathways. Computerised clinical decision support (CCDS) is effective in changing practice in other fields [3], [4], but there is little evidence about its costs and benefits in emergency care [5].

Falls in older people are a growing problem as populations age [6]. One in three adults aged 65 or older falls each year [7]. In the UK the prevention of falls in older people is a priority [8]. Though prevention strategies are effective, [9]reducing falls depends on early identification of people at high risk, and delivery of interventions across traditional service boundaries [10], now advocated by national and international guidelines [11], [12]. Calls to emergency ambulance services (999 calls) for falls contribute up to about 8% of the workload of Emergency Medical Services in the UK and internationally [13], [14]. Some 40% of these patients do not go to hospital [15], though alternative pathways are often lacking. Although non-conveyance of patients attended by emergency ambulances is known to be risky [13], [16], [17], we know little about how paramedics decide whether to convey. A US study recognised the pragmatic nature of the negotiation with patients whether to go to hospital [18]. In the UK qualitative studies have found that crew members base decisions on several factors including paramedic experience, training and intuition; time of call during shift; patient preference, home circumstances; and distance to receiving unit [19], [20]. New pathways of care are now being developed for patients attended by emergency paramedics, for older people who fall, as well as other patients who may not need immediate care at an Emergency Department [21].

Evidence from trials in community and emergency settings suggests care offered by multi-disciplinary falls services improves outcomes for patients [22], [23]. A recent Cochrane review of 19 trials with 9500 participants estimated that falls services reduce falls by 24% (95% CI 14% to 33%) [9]. Hence we need to investigate how best to achieve appropriate triage by emergency paramedics of patients who have fallen. The aim of this trial was to test the effectiveness, safety (or avoidance of ‘harm’), and cost-effectiveness of CCDS, a technological innovation for emergency paramedics to use in the care of older people who have fallen [24]. We use the term ‘effectiveness’ in reporting this pragmatic trial to indicate effects on processes and outcomes of care that are clinically important to patients or operationally important to service providers. Our principal outcomes reflect the mechanisms through which we theorised that this intervention could improve outcomes – through avoiding attendance at Emergency Departments and referral to alternative community-based falls services.

## Materials and Methods

The protocol for this trial and supporting CONSORT checklist are available as supporting information; see Checklist S1 and Protocol S1.

### Ethics statement

This cluster randomised trial was approved by the Multi-Centre Research Ethics Committee for Wales (08/MRE09/12), who sanctioned post-recruitment contact with a vulnerable population in an emergency setting, and inclusion of all patients who did not opt out of the study. Participants therefore did not have to give oral or written consent to participate in the trial during their emergency episode, but consented to follow up in response to information about the trial sent by participating ambulance services 7–10 days after their index event. Following processes agreed by the Ethics Committee, these services passed non-dissenting participants' contact details to the research team, and kept records of dissent in hard copy and electronically. Paramedics gave informed consent to participate. We registered the trial at: http://www.controlled-trials.com/ISRCTN10538608.

### Study design

Cluster trials are appropriate to evaluate interventions targeted at health professionals. Thus Support and Assessment for Fall Emergency Referrals 1 (SAFER 1) was a cluster trial with paramedics as the unit of randomisation. [24]

### Setting

We recruited patients at two UK study sites from November 2009 until October 2010. Delays in implementing a national information technology programme [25] reduced these from three to two: Site one, an urban centre where we recruited paramedics from four ambulance stations; and Site two, where we recruited paramedics from nine stations across a mixed urban and rural area.

### Participants

Paramedics were eligible to participate in SAFER 1 if they worked at any of 13 ambulance stations with a falls referral pathway in place; they continued to be eligible if they moved from one of these stations to another. In practice such a pathway requires a community-based falls service to accept direct referral of older people who fall by paramedics at the scene of their fall. Within an agreed space of time (typically within 1 week) falls services contact the patient and arrange a home visit to assess clinical and social needs and to arrange ongoing community based support [26]. The chief investigator invited all eligible paramedics to participate in SAFER 1 using local media to support recruitment. The trial team consented volunteers and passed anonymous details to the West Wales Organisation for Rigorous Trials in Health (WWORTH) for randomisation stratified by current ambulance station. Patients were eligible for SAFER 1 if they were: aged 65 or over; living in the catchment area of a participating falls service; and attended by a study paramedic following their first emergency call categorised by the call-taker as a fall during the study period. We excluded those living in nursing homes as they were not eligible for care from participating falls services.

### Interventions

The health technology evaluated in the experimental arm was CCDS on hand-held Tablet computers for use by paramedics to decide whether to take patients who had fallen to an Emergency Department or leave them at home with referral to a community-based falls service. Site one implemented the CCDS simultaneously with a system for electronic patient data capture; while Site two, where a different electronic data capture system was already in place, added CCDS software to the existing system. However neither site fully integrated CCDS with the electronic software; in particular Site one experienced many teething problems including loss of network signal and hardware failures. Control paramedics at both sites provided usual care, with paper-based protocols to assess patients and make decisions about their care, including patients who had fallen. Usual care comprised assessment, treatment on scene as required and default conveyance to the Emergency Department unless the patient refused to travel to hospital. Although we know that practice is variable, we did not attempt to standardise care in the control arm as there is little evidence about what is best for patients. Both groups could refer older people who had suffered a fall to community-based falls services.

### Outcomes

#### Principal individual outcomes

1. Effectiveness – proportion of participants left at scene without conveyance to an Emergency Department and proportion referred to falls services
2. Safety – proportion of participants with adverse events (harm) up to one month (999 call, Emergency Department attendance, emergency admission to hospital, or death);
3. Cost-effectiveness – comparison of costs of implementation of CCDS for paramedics and its benefits in the form of patient utility modelled over 12 months.

### Secondary individual outcomes

Self-reported falls; fall-related self-efficacy (‘fear of falling’) [27]; health-related quality of life (SF12) [28] and patient satisfaction (Quality of Care Monitor) [29] were gathered through postal questionnaires completed by patients or their carers. Operational indicators – ambulance service job cycle time, length of episode of emergency care and costs of care – were gathered from routine NHS sources. Though we had planned to include quality of clinical documentation, internal validation showed that the adoption of CCDS led to double data entry and risk of intervention bias. We explored implementation and adoption issues through focus groups and semi-structured interviews with practitioners, and reported the results elsewhere [30].

### Sample size

After redesigning SAFER 1 following delays in implementing the intervention, we powered it to detect clinically important changes in the proportion of participants who make another emergency call for a fall within a month (or die) – the ‘safety’ criterion. We calculated that a simple random sample of 622 participants would yield 80% power when using a 5% significance level to detect a fall in that proportion from 30% to 20%. To adjust for clustering by the 42 paramedics recruited (rather than the 13 ambulance stations at which they worked), we assumed that the intra-paramedic correlation coefficient (IPCC) was 0.02, and applied Donner's formula [31] to yield a target sample size of 865, namely 42 paramedics each recruiting an average of 20.6 participants or 622×[1+(20.6–1)×0.02].

### Randomisation

The West Wales Organisation for Rigorous Trials in Health (WWORTH) independently used random number tables to allocate paramedics, consented and stratified by current ambulance station, between intervention and control arms. It was possible to blind analysts to these allocations, but not paramedics or patients.

### Patient recruitment and data retrieval

**Figure 1:**
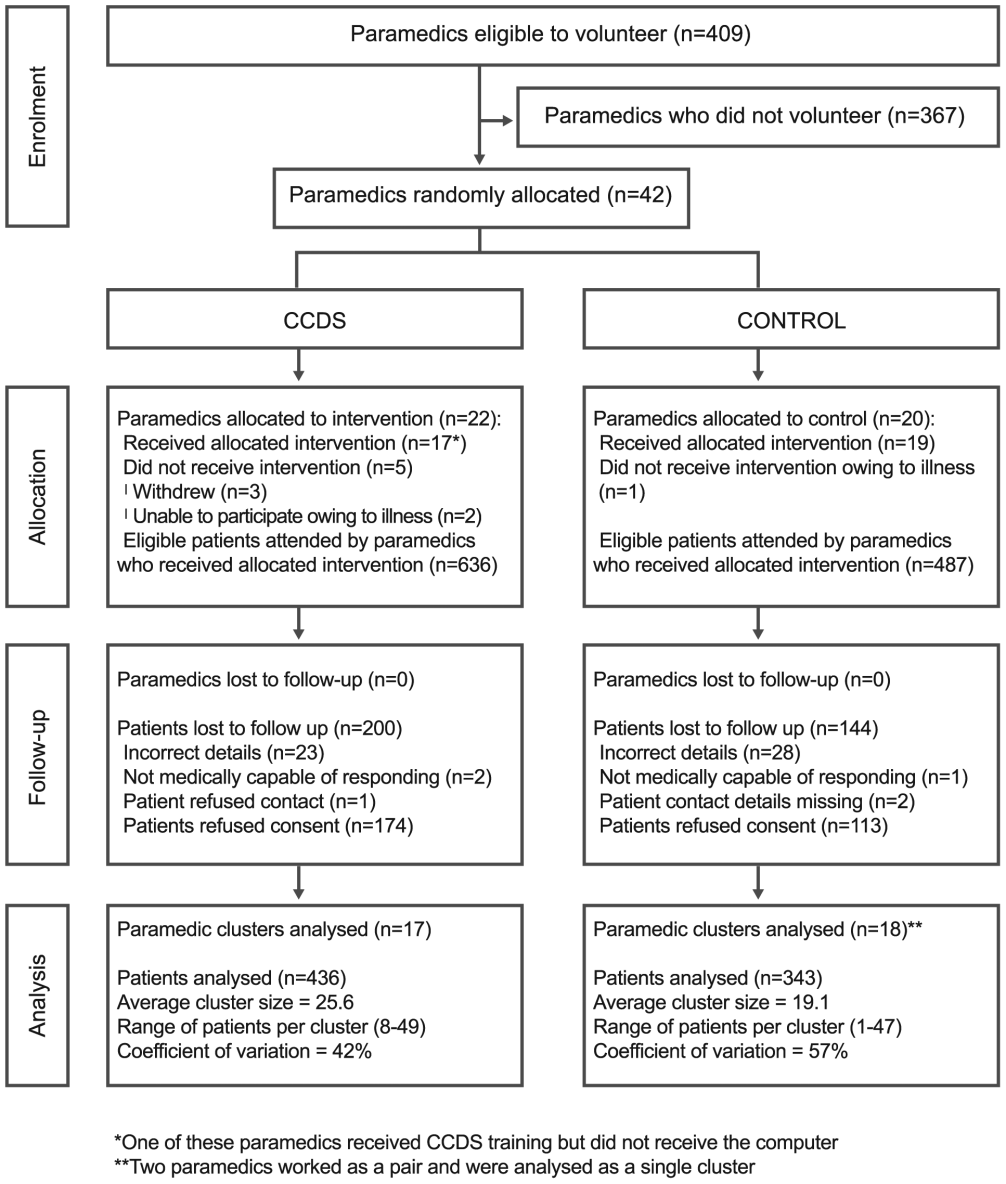
Flow of paramedics and patients through the trial.

Attending paramedics consented patients to treatment, but not trial participation owing to the emergency nature of the contact. Ambulance service staff identified potential participants from electronic records completed by control room staff, then confirmed eligibility from records completed by attending paramedics. They contacted participants by post within 10 days of the index call to give them the opportunity to opt out of follow up. At both sites we retrieved identifiable data about subsequent emergency calls and referrals to falls services and their outcomes from the ambulance services. Site one retrieved anonymised linked data about Emergency Department attendances, emergency hospital admissions and deaths from a central databank [32] although this process delayed analysis and reporting. At Site two we retrieved identifiable data about Emergency Department attendances and emergency admissions from individual National Health Service care providers; and about deaths from the Office of National Statistics. The flow of paramedics and patients through the trial is shown in Figure 1.

### Statistical methods

**Figure 2:**
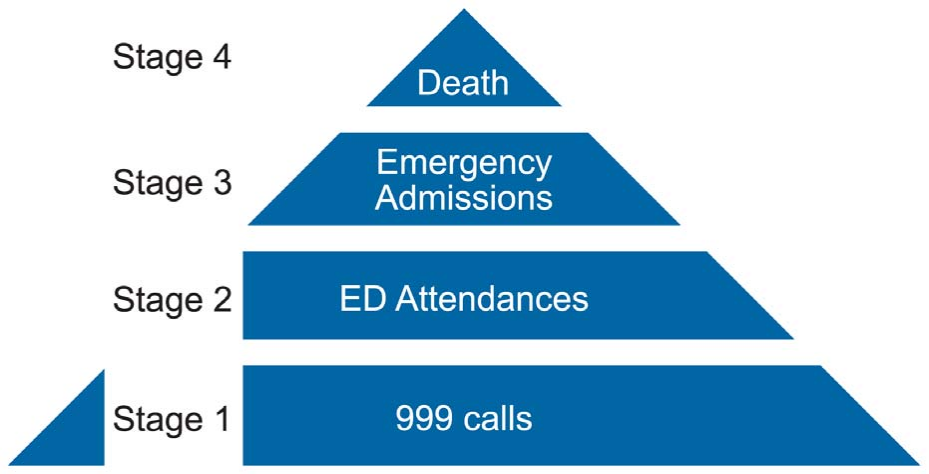
Hierarchy of harms.

In pre-specified analyses we used SPSS version 19 to fit multi-level logistic, linear and negative binomial regression models to, respectively, binary, measurement and count data available at one month on referrals to falls services, the hierarchy of ‘harms’ in Figure 2, and related outcomes, adjusting for statistically significant confounders, but not for multiple testing. Potential confounders included: ambulance service (site); patient's age, gender and distance to nearest Emergency Department; date of recruitment and whether call was out of hours. For secondary outcomes we again used multi-level models, adjusted for significant confounders, and imputed missing data, by published rules when available. Specifically, missing responses to individual SF12 questions were imputed using Expectation Maximisation methods [33] missing SF12-related scores were imputed using regression-based methods and set to zero on participant death. Similar regression-based methods impute missing ‘fear of falling’ and participant satisfaction scores.

To extend the outcomes of SAFER 1 to one year, we adopted a modelling approach similar to that used by Goitein to extend the outcomes of CT scans of gastric carcinoma to survival over time [34]. Specifically we inferred that participants referred to falls services in SAFER 1 would achieve the clinical outcomes reported for intervention participants with similar characteristics in the Community Falls Prevention Trial of referral to falls services [22]; and that participants not so referred would achieve the clinical outcomes reported for control participants with similar characteristics in the Community Falls Prevention Trial. In doing so we exploited the similarity of populations and outcomes between SAFER 1 and the Community Falls Prevention Trial, in particular by standardising by age and referral rates using Site two, where electronic data capture was already in use as the standard.

### Cost-effectiveness

We undertook a cost-effectiveness analysis from the perspective of the UK NHS and personal social services and used quality of life measured by the SF12, referrals to falls services, 999 calls, Emergency Department attendances and inpatient stays as outcomes over the next 30 days. To extend our time horizon to one year, we integrated the effects of CCDS on referrals to falls services as estimated by SAFER 1 with the effects of referrals to falls services on events over one year, especially patient utilities, as estimated by the earlier trial [22]. We used probabilistic sensitivity analysis to assess the extent to which the intervention gave value for money relative to using the same resources elsewhere. We estimated the implementation costs of CCDS, taking account of whether electronic data capture was already in place. We estimated the costs of staff, equipment and consumables from the ambulance services with and without existing electronic data capture; and the costs of healthcare use by multiplying that use by published unit costs (Table 1).

**Table 1:** NHS unit costs.

Health Service Resource	Unit cost (2009–10)	Source
Attendance by ambulance – conveyed	£246	NHS Reference Costs
Attendance by ambulance – not conveyed	£225	NHS Reference Costs
Attendance at Emergency Department	£399 to £445^a^	NHS Reference Costs
Inpatient stay per day	£237 to £414^a^	NHS Reference Costs
Referral to falls service	£77^b^	NHS Reference Costs

a Depending on level of treatment received

b Derived from discussions with Age Concern; equivalent to the unit cost of referral to ‘Hospital at Home or Early Discharge Schemes’

### Deviations from protocol

Deviations from the original study protocol: (1) We reduced paramedic training in consultation with participating ambulance services from two days to half a day including assessment of competence (2) We were unable to analyse some outcomes that varied between hospitals, for example categorisation of falls; we therefore analysed only the generic outcome ‘emergency admission to hospital’ (3) To reduce questionnaire length and maximise response rates, we did not collect costs incurred by participants, not least because in the UK they do not contribute financially to care provided by the falls services.(4) We did not measure outcomes at six months as planned, owing to delays in implementing the intervention.

## Results

### Recruitment, participant flow and questionnaire response rates

Eligible paramedics who volunteered for SAFER 1 numbered 27 out of 47 (57%) in Site one and 15 out of 362 (4%) in Site two. We allocated 22 paramedics at random to the intervention arm and 20 to control (Figure 2). Five paramedics in the intervention arm did not receive the intervention and were excluded from analysis, together with one in the control arm. However paramedics in the control arm attended fewer patients owing to long-term sickness, particularly in Site two, resulting in the recruitment of 343 controls compared with 436 in the intervention arm (Table 2). One intervention paramedic received training but no equipment; he remained in the intervention group for analysis by treatment allocated. We retrieved primary outcome data from routine sources for all 779 participants. Table 3 shows that 434 (61%) of those who did not opt out or die responded to postal questionnaires.

**Table 2:** Characteristics of participants recruited to Intervention and Control groups.

Characteristics of individual or cluster		Intervention	Control
		(n = 436)	(n = 343)
Men		153 (35%)	132 (39%)
Women		283 (65%)	211 (61%)
Median age in years (inter quartile range)		83 (77–89)	82 (76–88)
Site 1		235 (54%)	225 (66%)
Site 2		201 (46%)	118 (34%)
Made index call out of hours (%)		256 (59%)	189 (55%)
No of paramedics		17	19
No of patients attended by paramedics	Minimum	8	1
	Maximum	49	47
	Average	25.6	19.1

**Table 3:** Questionnaire response rates.

	Intervention		Control		Total
	Site 1	Site 2	Site 1	Site 2	
Eligible patients	235	201	225	118	779
Declined questionnaire follow up	2	10	5	16	33
Died	11	8	9	2	30
Questionnaire completed	129	117	123	65	434
Response rate	58.1%	63.9%	58.3%	65.0%	60.6%

### Principal effects up to one month – analysis by treatment allocated

CCDS usage was much lower in Site one, where CCDS and electronic patient data capture were both new (5/235 participants  = 2%), than in Site two, where electronic data capture was already in place (49/201 participants  = 24%). Patients attended by intervention paramedics were twice as likely to be referred to a falls service [42/436 (9.6%) compared with 17/343 (5.0%); OR 2.04, 95% CI 1.12 to 3.72], an effect that was consistent between sites. Non-conveyance rate was higher in the intervention group, but not significantly so [183/436 (42.0%) compared with 126/343 (36.7%); OR 1.13, 95% CI 0.84 to 1.52]. Table 4 shows that intervention and control groups did not differ across the hierarchy of outcomes (Figure 2); subsequent emergency healthcare contacts or death occurred in 155 of 436 intervention participants (35.6%) compared with 111 of 343 controls (32.4%) [OR 1.15, 95% CI 0.85 to 1.56]. Furthermore there was no difference between groups in participant-reported outcome measures.

**Table 4:** Outcomes at one month by treatment allocated – adjusted for significant covariates.^a^

	Raw data			95% CI			Intra-paramedic correlation	
Primary Outcomes	CCDS	Control	Adjusted comparison^b^	Lower	Upper	Sig Level	estimateb2	95% CI
Patients referred to falls service^c^	42 (9.6%)	17 (5.0%)	OR = 2.036	1.115	3.717	0.021	0.050	0.020, 0.092
Patients left at scene without conveyance to ED^d^	183 (42.0%)	126 (36.7%)	OR = 1.131	0.839	1.523	0.419	0.043	0.016, 0.084
Patients dying	19 (4.4%)	11 (3.2%)	OR = 1.375	0.645	2.930	0.409	0	Not applicable
Patients with further emergency admission to hospital or death^e^	69 (15.8%)	49 (14.3%)	OR = 1.129	0.757	1.685	0.552	0.029	0.010, 0.061
Patients with further emergency (ED attendance or emergency admission to hospital) or death^f^	92 (21.1%)	61 (17.8%)	OR = 1.296	0.899	1.870	0.165	0.018	0.004, 0.043
Patients with further emergency event (999 call, ED attendance, emergency admission to hospital) or death^g^	151 (34.6%)	100 (29.1%)	OR = 1.266	0.931	1.720	0.132	0.017	0.003, 0.044
Number of further emergency events per patient^h^ - mean	0.65	0.62	λ = 0.997	0.741	1.343	0.987	0.005	0.000, 0.027
SD	1.30	1.35						
	(n = 436)	(n = 343)						
**Secondary Outcomes**								
Patients who reported ≥1 further fall^i^	135/236 (57.2%)	112/175 (64.0%)	OR = 0.752	0.503	1.124	0.165	0.030	0.005, 0.081
Fall related self efficacy (fear of falling) – mean	3.74	4.08	Δ = −0.34	−0.85	0.17	0.187	0.014	0.001, 0.050
SD^j^	2.84	3.12						
	(n = 239)	(n = 177)						
**Quality of Life**								
SF12 MCS^k^- mean	41.9	42.9	Δ = −0.74	−2.83	1.28	0.457	0.017	0.001, 0.057
SD	10.3	10.9						
	(n = 239)	(n = 177)						
SF12 PCS^l^– mean	29.0	30.0	Δ = −0.13	−1.65	1.39	0.866	0	Not applicable
SD	8.0	8.5						
	(n = 239)	(n = 177)						
**Patient satisfaction**								
QC Technical – mean	97.8	98.2	Δ = −0.38	−2.42	1.67	0.719	0	Not applicable
SD	10.7	9.4						
	(n = 228)	(n = 165)						
QC Interpersonal^m^- mean	94.7	92.8	Δ = 1.43	−0.70	3.57	0.188	0.009	0.000, 0.058
SD	9.5	10.5						
	(n = 212)	(n = 155)						
Length of job cycle time (minutes)^n^– mean	91.0	80.6	Δ = 8.89	2.32	15.26	0.008	0.026	0.007, 0.057
SD	45.3	42.8						
	(n = 436)	(n = 341)						
Length of episode of care (minutes)^o^ – mean	227	240	Δ = −5.68	−38.5	27.2	0.734	0.079	0.038, 0.136
SD	216	227						
	(n = 359)	(n = 293)						

a Covariates considered: Group; site; their interaction; age (years); distance to ED (miles); date of recruitment (days since start of study); gender and whether the index call was ‘Out of (GP) Hours’.

b Comparison between CCDS and control groups reflects variable under consideration: Odds Ratio (OR) for logistic regression models for binary variables; multiplicative event rate ratio (λ, scalar) for negative binomial regression models for counts; and additive group effect (Δ, in same units as dependent variable) for linear models for continuous variables.

b2 Given consistently low intra-paramedic correlations in the primary participant based outcomes, corresponding terms are omitted from the fitted models.

**Key to statistically significant covariates:**

c Site (p = 0.007); age (p = 0.013); recruitment point (p<0.001)

d Site (p<0.001); out of hours (p = 0.017)

e Out of hours (p = 0.022); recruitment point (p = 0.020)

f Out of hours (p = 0.015); site (p = 0.004)

g Out of hours (p = 0.006)

h Out of hours (p = 0.001); days at risk (p = 0.002)

i Distance to ED (p = 0.046)

j Self-completed questionnaire (p<0.001)

k Self-completed questionnaire (p = 0.016)

l Gender (p = 0.004); self-completed questionnaire (p<0.001)

m Distance to ED (p = 0.049)

n Distance to ED (p = 0.028); out of hours (p = 0.001)

o Site (p<0.001)

Job cycle time for emergency ambulances was 8.9 minutes longer for intervention patients than for control patients (95% CI 2.32 to 15.26), although the total emergency episode of care (including time in Emergency Departments when participant were conveyed) was 5.7 minutes shorter (95% CI −38.5 to 27.2). Table 5 shows the costs of implementing CCDS with and without electronic data capture in place. Table 6 shows (non-significant) differences in healthcare resource use by one month. As SAFER 1 used SF12 to collect quality of life data over only 30 days, we exploited the earlier trial of referral to falls services [20] to extrapolate those findings beyond 30 days. This enabled us to use the EQ-5D (a standardised tool for measuring health outcomes) to estimate the utility gain due to the increase in referrals to the falls service as 0.0139 (95% CI −0.0361 to 0.0638). When we combined this with the incremental cost of £208 appropriate to existing electronic data capture software, (Table 7) the estimated cost per quality-adjusted life-year (QALY) was £14,964 (lower 2.5% confidence bound £3260); when we combined it with the incremental cost of £308 in the absence of electronic data capture software,(Table 7) the estimated cost per QALY was £22,154 (lower 2.5% confidence bound £4828). The UK National Institute of Health and Care Excellence (NICE) generally recommends that the UK National Health Service buy treatments that cost less than £20,000 to £30,000 to gain one QALY. The probabilities that our estimates fall below the NICE thresholds are 58% (existing software, below £20,000), 61% (existing software, below £30,000), 40% (no software, below £20,000) and 48% (no software, below £30,000) [35].

**Table 5:** Annual cost of implementing CCDS.

Cost category	Cost component	Description	Unit cost	Total cost	Unit cost	Total cost
			Site 1: CCDS & electronic data capture	Site 1: CCDS & Electronic data capture	Site 2: CCDS only	Site 2: CCDS only
Staff time	Project manager	50% time on project	£26,000	£13,000	£26,000	£13,000
	Training costs	Training of ‘trainers’ and paramedics for 6 hours	£12,355	£12,355	£12,355	£12,355
	IT support	One day per week	£100 per day	£5,000	£100 per day	£5,000
	Auditing	Project set up	£200	£200		
Equipment	12 hand held tablet PCs	For computerised clinical decision support	£3,850 per tablet^a^	£16,490		
	12 printers		£595 per printer^a^	£2,549		
	12 chargers		£25 per charger^a^	£107		
	Adapt vehicles to electronic data capture	Time to adapt vehicles	£60 per hour	£8,709		
		Engineering fee	£6.965			
	SIM cards		£38 per month	£5,472		
Consumables	Paper rolls	For printers	20 rolls/printer ×£6	£1,440	20 rolls/printer ×£6	£1,440
	Software licence			£396		£396
Other	Plain Healthcare	Technical support		£9.950		
Total cost (436 vehicles dispatched)				£75,668		£32,191
Average cost per vehicle dispatched				£174		£74

a Assuming the 12 sets of tablets, printers and chargers need replacing every 3 years; and converted to annualised capital charges using annual discount rate of 3.5%.

**Table 6:** Cost of implementing CCDS by one month.

	Intervention				Control				
	Number	Mean cost (n = 436)	SD of cost	Mean days in hospital	Number	Mean cost (n = 343)	SD of cost	Mean days in hospital	Mean cost difference (95% CI)
Implementing CCDS [+ electronic data capture if needed]		£74 [+£100]							£74 [+£100]
Initial 999 call	436	£234	£10	-	343	£233	£10		£1 (−£1, £3)
Initial ED attendance	215	£211	£214	-	179	£223	£214	-	−£12 (−£42, £18)
Initial hospital stay	140	£1,503	£2.961	1.84	104	£1,534	£3,050	1.22	−£31 (−£457, £394)
Subsequent 999 calls	174	£96	£241	-	127	£88	£218	-	£8 (−£25, £41)
Subsequent ED attendance	53	£52	£146	-	38	£47	£153	-	£5 (−£16, £26)
Subsequent hospital stay	58	£596	£1,933	4.49	46	£397	£1,338	4.58	£199 (−£31, £430)
Falls service referrals	42	£7	£23	-	17	£4	£17	-	£3 (£1, £6)
Total cost [+ electronic data capture]		£2,773 [+£100]	£3,527	-	-	£2,526	£3,435	-	£247 (−£247, £741) [+£100]

**Table 7:** Inferred net costs and consequences by 12 months.

Intervention group minus controls	Inferred from Logan et al [15]				Inferred from Logan [15] with Site 1 standardised to Site 2					
	Mean	95% confidence interval		Significance level	Mean	95% confidence interval		Significance level	Unit cost	Amount saved per patient year
Quality adjusted life years	0.0090	−0.0408	0.0588	0.723	0.0139	−0.0361	0.0638	0.587		
Self reported fall rate	−0.22	−1.33	0.89	0.700	−0.28	−1.38	0.83	0.626	£77^a^	£22
999 call rate	−0.14	−1.10	0.83	0.783	−0.19	−1.16	0.77	0.693	£235^a^	£45
ED presentation rate	−0.07	−0.42	0.29	0.717	0.08	−0.44	0.27	0.652	£422^a^	£34
Hospital admission rate	−0.002	−0.174	0.170	0.983	0.004	−0.168	0.175	0.967		
Mean number of inpatient bed days	0.15	−2.52	2.82	0.912	0.19	−2.48	2.86	0.889	£325^a^	−£62
Net resources saved by CCDS										£39
Net cost resources saved by CCDS										£308^b^ £208^c^

a From Table 1 – point estimate or mid-point of range

b Including £100 to implement electronic data capture

c When electronic data capture already in place

### Patterns and effects of CCDS usage

Individual intervention paramedics used CCDS between 0 and 22 times. This 22 accounted for 47% of the cases attended by this paramedic. The use of CCDS increased the proportion of patients referred to falls services [12/54 (22.2%) compared with 30/382 (7.9%); OR 3.35, 95% CI 1.60 to 7.04], and those not conveyed to the Emergency Department [35/54 (64.8%) compared with 126/343 (38.7%); OR 2.91, 95% CI 1.61 to 5.28] with no increase in harms [17/54 (31.5%) compared with 134/382 (35.1%); OR 0.85, 95% CI 0.46 to 1.58] (Table 8). Job cycle time was 6.4 minutes longer in patients when CCDS was used [96.6 minutes compared with 90.2 minutes; 95% CI for difference 6.6 to 19.3 minutes] but the whole emergency episode was 113.8 minutes shorter [126.6 minutes compared with 240.5 minutes; 95% CI for difference 45.6 to 182.1 minutes] (Table 9).

**Table 8:** Characteristics and primary outcomes at one month patients by treatment available and received.

		Raw data			95% CI				95% CI		
Patient characteristics	A: CCDS used	B: CCDS available but not used	C: CCDS not available	Comparison (A & B)^a^	Lower	Upper	Sig Level	Comparison (B&C)	Lower	Upper	Sig Level
Age (years) – mean	85.4	81.7	81.0	Δ_A_ = 3•64	1.37	5.90	0.002	Δ_x_ = −0.76	−1.92	0.40	0.197
SD	7.5	7.8	8.1								
	(n = 54)	(n = 382)	(n = 343)								
Proportion of females	36/54 (66.7%)	246/381 (64.6%)	211/342 (61.7%)	OR_A_ = 1.098	0.600	2.007	0.762	OR_C_ = 0.884	0.653	1.196	0.424
Distance to ED (Miles) – mean	11.5	9.5	7.8	Δ_A_ = 1.99	0.15	3.83	0.034	Δ_x_ = −1.69	−2.63	−0.74	<0.001
SD	7.3	7.3	4.7								
	(n = 52)	(n = 372)	(n = 324)								
Index call recorded outside GPs standard hours	35/54 (64.8%)	220/382 (57.6%)	185/343 (53.9%)	OR_A_ = 1.356	0.749	2.457	0.315	OR_C_ = 0.862	0.643	1.157	0.322
Date of recruitment (days since start of study) – mean, SD	182.5	196.4	188.8	Δ_A_ = −13.9	−39.1	11.4	0.281	Δ_X_ = −7.5	−20.4	5.4	0.254
SD	86.1	87.6	89.8								
	(n = 54)	(n = 382)	(n = 343)								
**Primary Outcomes**											
Patients referred to falls service	12/54 (22.2%)	30/382 (7.9%)	17/343 (5.0%)	OR_A_ = 3.352	1.596	7.040	0.001	OR_C_ = 0.612	0.331	1.130	0.117
Patients left at scene without conveyance to ED	35/54 (64.8%)	126/343 (36.7%)	126/343 (36.7%)	OR_A_ = 2.913	1.606	5.282	<0•001	OR_C_ = 0.918	0.679	1.240	0.578
Patients with further emergency event (999 call, ED attendance, emergency admission to hospital) or death	17/54 (31.5%)	134/382 (35.1%)	100/343 (29.2%)	OR_A_ = 0.850	0.461	1.567	0.603	OR_C_ = 0.762	0.557	1.042	0.089
Number of emergency events per patient – mean	0.56	0.67	0.62	Λ_A_ = 0•894	0.614	1.302	0.560	λ_x_ = 0.949	0.783	1.150	0.594
SD	1.14	1.32	1.35								
	(n = 54)	(n = 382)	(n = 343)								

a Comparison of groups uses group B as the reference, reflects the variable under consideration:

Odds Ratio (OR) for logistic regression models for binary variables;

Multiplicative Event Rate Ratio (λ, scalar) for negative binomial regression models for counts; and Additive Group Effect (Δ, in same units as dependent variable) for linear models for continuous variables.

**Table 9:** Secondary outcomes at one month patients by treatment available and received.

		Raw data			95% CI				95% CI		
Patient characteristics	A: CCDS used	B: CCDS available but not used	C: CCDS not available	Comparison (A & B)^a^	Lower	Upper	Sig Level	Comparison (B&C)	Lower	Upper	Sig Level
**Secondary outcomes**											
Patients who reported one or more further falls	15/26 (57.7%)	117/206 (56.8%)	109/169 (64.5)	OR_A_ = 1.037	0.454	2.368	0.931	OR_C_ = 1.382	0.909	2.100	0.130
Fall-related self efficacy (Fear of falling) – mean	3.22	3.46	4.08	Δ_A_ = −0.25	−1.46	0.97	0.690	Δ_x_ = 0.61	0.02	1.21	0.043
SD	2.68	2.87	3.12								
	(n = 26)	(n = 213)	(n = 177)								
Quality of life: SF-12 MCS – mean	43.47	41.72	42.94	Δ_A_ = 1.75	−2.56	6.07	0.425	Δ_x_ = 1.23	−0.89	3.34	0.255
SD	9.70	10.39	10.88								
	(n = 26)	(n = 213)	(n = 177)								
SF-12 PCS – mean	29.70	28.97	29.96	Δ_A_ = 0.73	−2.62	4.08	0.669	Δ_x_ = 0.98	−0.66	2.62	0.239
SD	7.01	8.11	8.47								
	(n = 26)	(n = 213)	(n = 177)								
Patient satisfaction											
QC Technical – mean	100, 0	97.52	98.18	Δ_A_ = 2.48	−1.70	6.65	0.244	Δ_x_ = 0.66	−1.44	2.76	0.539
SD	0	11.37	9.39								
	(n = 26)	(n = 202)	(n = 165)								
QC Interpersonal – mean, SD	95.42	94.57	92.84	Δ_A_ = 0.84	−3.39	5.07	0.696	Δ_x_ = −1.74	−3.85	0.38	0.108
	7.21	9.72	10.49								
	(n = 24)	(n = 188)	(n = 155)								
Length of job cycle time (minutes) – mean	96.6	90.3	80.6	Δ_A_ = 6.4	−6.2	19.0	0.321	Δ_x_ = −9.7	−16.1	−3.2	0.003
SD	36.5	46.4	42.8								
	(n = 54)	(n = 382)	(n = 341)								
Length of episode of care (minutes) – mean	126.6	240.5	240.3	Δ_A_ = −113.8	−184.0	−43.7	0.001	Δ_x_ = −0.2	−35.2	34.8	0.992
SD	104.2	224.0	227.0								
	(n = 43)	(n = 315)	(n = 293)								

a Comparison of groups uses group B as the reference, reflects the variable under consideration:

Odds Ratio (OR) for logistic regression models for binary variables; and Additive Group Effect (Δ, in same units as dependent variable) for linear models for continuous variables.

### Adverse events

We initiated the procedure for investigating a Suspected Unexpected Serious Adverse Reaction (SUSAR) only once – following the death of a trial participant left at home by the attending crew with a referral to the falls service. The ambulance service principal investigator (RW) reported formally to the independent Data Monitoring and Ethics Committee and Trial Steering Committee that this incident occurred in the control arm of the trial and the chairs of the two committees agreed to take no further action.

### Data sharing

Some participants gave informed consent for data sharing; their data are available from h.a.snooks@swansea.ac.uk. However information governance does not allow access to the unconsented patient data held in the Secure Anonymised Information Linkage databank. For the Community Falls Prevention Trial [22] the technical appendix, statistical code, and dataset are available from pip.logan@nottingham.ac.uk. The technical report of SAFER 1 is available at: www.ictri.port.ac.uk/projects2/reports/H%20Snooks%20final%20report%20Nov%202011.pdf.

## Discussion

### Principal findings

CCDS usage was low, but the proportion of patients referred to falls services was twice as high in the intervention group as in controls. We found no differences between intervention and control groups in subsequent ‘harms’, patient-reported quality of life, satisfaction or fear of falling at one month. Job cycle time was nine minutes longer for intervention patients. By integrating these findings with those of the randomised trial evaluating referral to community falls services in a similar population [22], we found that CCDS was potentially cost-effective when complementing an existing electronic data capture system.

### Strengths and limitations

We conducted a systematic review of the use of computerised clinical decision support (CCDS) in the emergency care setting. Though this search led us to 20 primary studies or reviews of the effectiveness of CCDS, we identified no other study of CCDS in pre-hospital emergency care. Together these studies of the effectiveness of CCDS across many fields show positive effects on processes of care, including improved compliance with guidelines and reduced time between presentation of problem and the start of definitive care, but reports of low CCDS usage were common.

Key strengths of this study lie in the rigorous conduct of a randomised trial across 13 representative ambulance stations and integration of findings with a second trial previously undertaken in four representative Primary Care Trusts [22]. SAFER 1 suffered from three main limitations: quality of operational data; foreshortening of data collection; and infrequent use of CCDS. First data on Emergency Department attendances, inpatient admissions and mortality were initially incomplete, owing to poor recording of patient identifiable data during the initial episode, and difficulty in matching them to central registers. Fortunately the cumulative nature of our primary outcome, and the manual searching of 999 records for subsequent events, generated a primary outcome for all trial participants. Secondly implementation of the randomised trial, known to be challenging in pre-hospital emergency care [36], was generally successful. However delays in implementing CCDS prevented collection of the planned six-month outcomes within the funded period. Hence the main patient outcome, at one month, addressed the safety of the intervention more than its clinical effectiveness. Falls services do not offer crisis intervention, but longer-term multi-disciplinary assessment and tailored care [10]; hence referral to these services is unlikely to yield benefits within a month. Thus the SAFER 1 trial reports best on harms following the index call, and referrals to falls services. Fortunately we were able to translate our findings on referrals into outcomes over 12 months by integrating them with those of the Community Falls Prevention Trial of referrals to falls services [22]. Though the populations and outcomes of the two integrated trials were similar, there is a danger that practical integration of CCDS and falls services would have proved more difficult in two distinct sites than integrating two data sets through computer modelling. Finally technical problems affected CCDS performance in Site one. Fortunately use was higher in Site two, where paramedics were already using the hardware to document patient care. Though recruiting 779 participants against a target of 865 was another potential weakness, the actual intra-paramedic correlation coefficient of 0.017 (Table 4) was less than the 0.02 we had assumed, with the result that the power of SAFER 1 fell by less than 2%.

### Implications for policy, practice and research

Though we were able to ameliorate the first two limitations, the fact that paramedics used CCDS for only one eighth of trial participants reduced its potential cost-effectiveness. Even so, increased referrals to falls services occurred more widely than in participants for whom CCDS was used, suggesting that this complex intervention affected practice generally, perhaps through learning from training or CCDS use. Decision-making is complex in pre-hospital emergency care. Although the trial called ‘Effectiveness of paramedic practitioners in attending 999 calls from elderly people in the community' reported effective changes in paramedic care [37], paramedic interventions have often found difficulty in changing practice [38]. There is evidence that crews use protocols to justify current practice rather than to inform decisions: they decide to leave the patient at home and then use the protocol to justify this decision [39]. Our qualitative data will enable us to explore these issues. Operational data showed that job cycle time increased for patients attended by intervention paramedics. Some of this increase may have been due to lack of familiarity or infrequent use. Other problems arose from the introduction at Site one of an entire electronic data capture system, known initially to increase time on scene [40]. Integration of electronic data capture and CCDS software may ameliorate these. Furthermore reduced length of episode in the intervention group suggests that avoided journeys and reduced time in the Emergency Department may offset the increased pre-hospital phase. Thus our findings show that CCDS could have an important role to play in the provision of safe and effective care for this frail but growing patient group.

Given the infrequent use of CCDS in this trial, and the time needed to detect benefits of increased referrals to falls services, we did not expect changes in health outcomes within 30 days, the span of the SAFER 1 trial. However the finding that referrals to falls services doubled, the inference of positive health outcomes at one year from the previous trial, and the low cost of the intervention suggest that CCDS has the potential to become cost-effective in the pre-hospital management of falls. To confirm these early findings needs further research, especially with integrated software and longer term outcomes.

## Conclusion

Computerisation of health records is advancing in the UK [41], and abroad [42]. Many ambulance services have implemented electronic data capture, linking dispatch information to records completed on scene by ambulance crews and ED records. Thus the main findings of SAFER 1 – that CCDS is safe, effective in referring older people who fall to community falls services and potentially cost effective – are encouraging. These preliminary findings lead us to recommend evaluating CCDS within an integrated system for the care of patients who fall or otherwise do not need immediate care at the Emergency Department.

## Supporting Information

Checklist S1 **CONSORT checklists for PLOS ONE.**
(DOCX)

File S1 **Participant invitation letter (opt out consent).**
(DOCX)

File S2 **SAFER 1 Patient Information sheet.**
(DOCX)

File S3 **SAFER 1 Patient Questionnaire at one month.**
(DOCX)

Protocol S1 **SAFER protocol 2008.**
(DOC)
