# Supplementary material for: Support and Assessment for Fall Emergency Referrals (SAFER 1): Cluster Randomised Trial of Computerised Clinical Decision Support for Paramedics
Source: PLoS One. 2014 Sep 12;9(9):e106436. doi: 10.1371/journal.pone.0106436 (PMC4162545; doi:10.1371/journal.pone.0106436)
Supplement: Checklist S1 — CONSORT checklists for PLOS ONE. (DOCX) [file pone.0106436.s001.docx]

**CONSORT Checklist**

**Table 1: CONSORT 2010 checklist with extensions for CRTs, PROs and Harms**

| Section/Topic | Item no | Standard checklist item | |  | Extension for cluster designs |  | Extension for PROs |  | Extension for Harms | Section |
| --- | --- | --- | --- | --- | --- | --- | --- | --- | --- | --- |
|  | | | Title and abstract | | |  |  |  |  |  |
|  | 1a | Identification as a randomised trial in the title | |  | Identification as a cluster randomised trial in the title |  |  |  | If the study collected data on harms and benefits, the title or abstract should so state | Title |
|  | 1b | Structured summary of trial design, methods, results, and conclusions (for specific guidance see CONSORT for abstracts) | |  |  |  | The PRO should be identified in the abstract as a primary or secondary outcome |  |  | Abstract |
|  | | | Introduction | | |  |  |  |  |  |
| Background and objectives | 2a | Scientific background and explanation of rationale | |  | Rationale for using a cluster design |  | Including background and rationale for PRO assessment |  | If the trial addresses both harms and benefits, the introduction should so state | Introduction & Study design |
|  | 2b | Specific objectives or hypotheses | |  | Whether objectives pertain to the cluster level, the individual participant level or both |  | The PRO hypothesis should be stated and relevant domains identified if applicable |  |  | Introduction and Study design |
|  | | | Methods | | |  |  |  |  |  |
| Trial design | 3a | Description of trial design (such as parallel, factorial) including allocation ratio | |  | Definition of cluster and description of how the design features apply to the clusters |  |  |  |  | Study design, Participants & Interventions |
|  | 3b | Important changes to methods after trial commencement (such as eligibility criteria), with reasons | |  |  |  |  |  |  | Deviations from protocol |
| Participants | 4a | Eligibility criteria for participants | |  | Eligibility criteria for clusters |  | Not PRO specific, unless the PROs were used in eligibility or stratification criteria |  |  | Participants |
|  | 4b | Settings and locations where the data were collected | |  |  |  |  |  |  | Setting |
| Interventions | 5 | The interventions for each group with sufficient details to allow replication, including how and when they were actually administered | |  | Whether interventions pertain to the cluster level, the individual participant level or both |  |  |  |  | Interventions |
| Outcomes | 6a | Completely defined pre-specified primary and secondary outcome measures, including how and when they were assessed | |  | Whether outcome measures pertain to the cluster level, the individual participant level or both |  | Evidence of PRO instrument validity and reliability should be provided or cited if available including the person completing the PRO and methods of data collection |  | List addressed adverse events with definitions for each  Clarify how harms-related information was collected | Outcomes & Adverse events  Patient recruitment and data retrieval |
|  | 6b | Any changes to trial outcomes after the trial commenced, with reasons | |  |  |  |  |  |  | Secondary individual outcomes |
| Sample size | 7a | How sample size was determined | |  | Method of calculation, number of clusters(s) (and whether equal or unequal cluster sizes are assumed), cluster size, a coefficient of intracluster correlation (ICC or *k*), and an indication of its uncertainty |  | Not required for PRO unless it is a primary study outcome |  |  | Sample size |
|  | 7b | When applicable, explanation of any interim analyses and stopping guidelines | |  |  |  |  |  |  | n/a |
| Randomisation:  Sequence generation | 8a | Method used to generate the random allocation sequence | |  |  |  |  |  |  | Randomisation |
|  | 8b | Type of randomisation; details of any restriction (such as blocking and block size) | |  | Details of stratification or matching if used |  |  |  |  | Randomisation |
| Randomisation:  Allocation concealment mechanism | 9 | Mechanism used to implement the random allocation sequence (such as sequentially numbered containers), describing any steps taken to conceal the sequence until interventions were assigned | |  | Specification that allocation was based on clusters rather than individuals and whether allocation concealment (if any) was at the cluster level, the individual participant level or both |  |  |  |  | Randomisation |
| Randomisation:  Implementation | 10 | Who generated the random allocation sequence, who enrolled participants, and who assigned participants to interventions | |  | Replace by 10a, 10b and 10c |  |  |  |  | Randomisation |
|  | 10a |  | |  | Who generated the random allocation sequence, who enrolled clusters, and who assigned clusters to interventions |  |  |  |  | Randomisation |
|  | 10b |  | |  | Mechanism by which individual participants were included in clusters for the purposes of the trial (such as complete enumeration, random sampling) |  |  |  |  | Randomisation |
|  | 10c |  | |  | From whom consent was sought (representatives of the cluster, or individual cluster members, or both), and whether consent was sought before or after randomisation |  |  |  |  | Participants |
| Blinding | 11a | If done, who was blinded after assignment to interventions (for example, participants, care providers, those assessing outcomes) and how | |  |  |  |  |  |  | Randomisation |
|  | 11b | If relevant, description of the similarity of interventions | |  |  |  |  |  |  | n/a |
| Statistical methods | 12a | Statistical methods used to compare groups for primary and secondary outcomes | |  | How clustering was taken into account |  | Statistical approaches for dealing with missing data are explicitly stated |  | Describe plans for presenting and analysing information on harms | Statistical methods |
|  | 12b | Methods for additional analyses, such as subgroup analyses and adjusted analyses | |  |  |  |  |  |  | Statistical methods |
|  | | | Results | | |  |  |  |  |  |
| Participant flow (a diagram is strongly recommended) | 13a | For each group, the numbers of participants who were randomly assigned, received intended treatment, and were analysed for the primary outcome | |  | For each group, the numbers of clusters that were randomly assigned, received intended treatment, and were analysed for the primary outcome |  | The number of PRO outcome data at baseline and at subsequent time points should be made transparent |  | Describe for each arm the participant withdrawals that are due to harms and their experiences with the allocated treatment | See CONSORT flowchart |
|  | 13b | For each group, losses and exclusions after randomisation, together with reasons | |  |  |  |  |  |  | See CONSORT flowchart |
| Recruitment | 14a | Dates defining the periods of recruitment and follow-up | |  |  |  |  |  |  | Patient recruitment and data retrieval |
|  | 14b | Why the trial ended or was stopped | |  |  |  |  |  |  | n/a |
| Baseline data | 15 | A table showing baseline demographic and clinical characteristics for each group | |  | Baseline characteristics for the individual and cluster levels as applicable for each group |  | Including baseline PRO data when collected | n/a |  | Tables 2 & 8 |
| Numbers analysed | 16 | For each group, number of participants (denominator) included in each analysis and whether the analysis was by original assigned groups | |  | For each group, number of clusters included in each analysis |  | Required for PRO results |  | Provide the denominators for analyses on harms | Recruitment, participant flow and questionnaire response rates |
| Outcomes and estimation | 17a | For each primary and secondary outcome, results for each group, and the estimated effect size and its precision (such as 95% confidence interval) | |  | Results at the individual or cluster level as applicable and a coefficient of intracluster correlation (ICC or k) for each primary outcome |  | For multidimensional PRO results from each domain and time point |  | Present the absolute risk per arm and per adverse event type, grade, and seriousness, and present appropriate metrics for recurrent events, continuous variables, and scale variables, whenever pertinent (refers to items 17, 18, 19)  Describe any subgroup analyses and exploratory analyses for harms (refers to items 17, 18, 19) | Principal effects up to one month & Patterns and effects of CCDS usage  See Figure 2 |
|  | 17b | For binary outcomes, presentation of both absolute and relative effect sizes is recommended | |  |  |  |  |  |  | Principal effects up to one month & Patterns and effects of CCDS usage |
| Ancillary analyses | 18 | Results of any other analyses performed, including subgroup analyses and adjusted analyses, distinguishing pre-specified from exploratory | |  |  |  | Including PRO analyses, where relevant | 6/7 |  | Principal effects up to one month & Patterns and effects of CCDS usage |
| Harms | 19 | All important harms or unintended effects in each group | |  |  |  |  |  |  | Adverse events |
|  | | | Discussion | | |  |  |  |  |  |
| Limitations | 20 | Trial limitations, addressing sources of potential bias, imprecision, and, if relevant, multiplicity of analyses | |  |  |  | PRO-specific limitations and implications for generalisabililty and clinical practice |  | Provide a balanced discussion of benefits and harms with emphasis on study limitations, generalisability, and other sources of information on harms (refers to items 20,21,22) | Strengths and limitations |
| Generalisability | 21 | Generalisability (external validity, applicability) of the trial findings | |  | Generalisability to clusters and/or individual participants (as relevant) |  |  |  |  | Strengths and limitations & Implications for policy, practice and research |
| Interpretation | 22 | Interpretation consistent with results, balancing benefits and harms, and considering other relevant evidence | |  |  |  | PRO data should be interpreted in relation to clinical outcomes including survival data where relevant |  |  | Implications for policy, practice and research & Conclusion |
| Other information | | | |  |  |  |  |  |  |  |
| Registration | 23 | Registration number and name of trial registry | |  |  |  |  |  |  | Submitted as supplemental information to publisher |
| Protocol | 24 | Where the full trial protocol can be accessed, if available | |  |  |  |  |  |  | Submitted as supplemental information to publisher |
| Funding | 25 | Sources of funding and other support (such as supply of drugs), role of funders | |  |  |  |  |  |  | Submitted as supplemental information to publisher |
