# Supplementary material for: Support and Assessment for Fall Emergency Referrals (SAFER 1): Cluster Randomised Trial of Computerised Clinical Decision Support for Paramedics
Source: PLoS One. 2014 Sep 12;9(9):e106436. doi: 10.1371/journal.pone.0106436 (PMC4162545; doi:10.1371/journal.pone.0106436)
Supplement: File S1 — Participant invitation letter (opt out consent). (DOCX) [file pone.0106436.s002.docx]

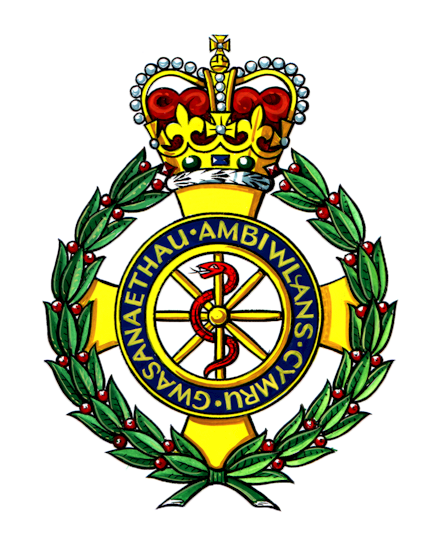


***Ymddiriedolaeth GIG Gwasanaethau Ambiwlans Cymru***

***Welsh Ambulance Services NHS Trust***

*Date*

Dear

We are currently carrying out a study with Swansea University looking at how to improve the emergency care of older people, called the SAFER1 trial.

Following your recent 999 call we would like to track your medical records as part of this study in order to include details of any injuries you have sustained, any further problems you have had and the care you have received. We would also like to send you a questionnaire about how you are feeling one month after your 999 call. We hope that you or your carer will complete this questionnaire so that we can include information about your health and experience of care in the study. We enclose an information sheet for you to read. This explains what being included would mean to you. We have also included a reply slip. Please read these carefully.

If you decide you want to be included in the study no further action is required at this point. However, if you would rather NOT be included, please tick one or both of the boxes on the reply slip and return it in the FREEPOST envelope provided as soon as you can.

If you have any questions, please contact the Study Co-ordinator, Antonio Sanchez on 01792 295385.

Thank you for taking the time to read this letter.

Yours sincerely,

Richard Whitfield Professor Helen Snooks

Practice Research & Development Manager School of Medicine

Welsh Ambulance Services NHS Trust Swansea University


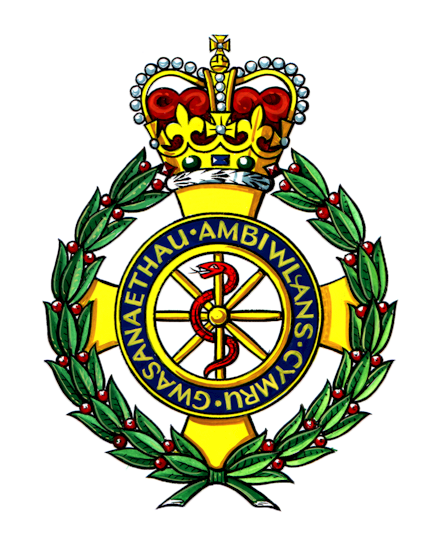


***Ymddiriedolaeth GIG Gwasanaethau Ambiwlans Cymru***

***Welsh Ambulance Services NHS Trust***

*Dyddiad*

Annwyl

Rydym wrthi ar hyn o bryd yn cynnal astudiaeth ar y cyd â Phrifysgol Abertawe er mwyn gweld sut y gellir gwella’r gofal brys a gaiff pobl hŷn. Caiff yr astudiaeth ei galw’n astudiaeth SAFER1.

Gan i chi ffonio 999 yn ddiweddar, hoffem olrhain eich cofnodion meddygol yn rhan o’r astudiaeth hon er mwyn cynnwys manylion unrhyw anafiadau a gawsoch, unrhyw broblemau eraill a gawsoch a’r gofal a gawsoch. Yn ogystal, hoffem anfon holiadur atoch i ganfod sut yr ydych yn teimlo fis ar ôl i chi ffonio 999. Rydym yn gobeithio y byddwch chi neu’ch gofalwr yn fodlon llenwi’r holiadur fel y gallwn gynnwys gwybodaeth am eich iechyd a’ch profiad o ofal yn yr astudiaeth. Rydym yn amgáu taflen wybodaeth i chi ei darllen. Mae’n esbonio beth fyddai cael eich cynnwys yn yr astudiaeth yn ei olygu i chi. Rydym wedi amgáu slip ateb hefyd. Dylech ddarllen y rhain yn ofalus.

Os byddwch chi’n penderfynu yr hoffech chi gael eich cynnwys yn yr astudiaeth, ni fydd angen i chi wneud unrhyw beth arall am y tro. Fodd bynnag, os byddai’n well gennych BEIDIO â chael eich cynnwys ynddi, ticiwch y naill flwch neu’r llall neu’r ddau flwch ar y slip ateb, ac anfonwch ef yn ôl atom cyn gynted ag y bo modd yn yr amlen RHADBOST a ddarparwyd.

Mae croeso i chi gysylltu â chydlynydd yr astudiaeth os oes gennych unrhyw gwestiynau, Antonio Sanchez, ar 01792 295385.

Diolch am neilltuo amser i ddarllen y llythyr hwn.

Yn gywir,

Richard Whitfield Yr Athro Helen Snooks

Rheolwr Ymchwil a Datblygu Ymarfer Ysgol Feddygaeth

Ymddiriedolaeth GIG Prifysgol Abertawe

Gwasanaethau Ambiwlans Cymru
