# Supplementary material for: Support and Assessment for Fall Emergency Referrals (SAFER 1): Cluster Randomised Trial of Computerised Clinical Decision Support for Paramedics
Source: PLoS One. 2014 Sep 12;9(9):e106436. doi: 10.1371/journal.pone.0106436 (PMC4162545; doi:10.1371/journal.pone.0106436)
Supplement: File S2 — SAFER 1 Patient Information sheet. (DOCX) [file pone.0106436.s003.docx]

SAFER 1 **Information Sheets**


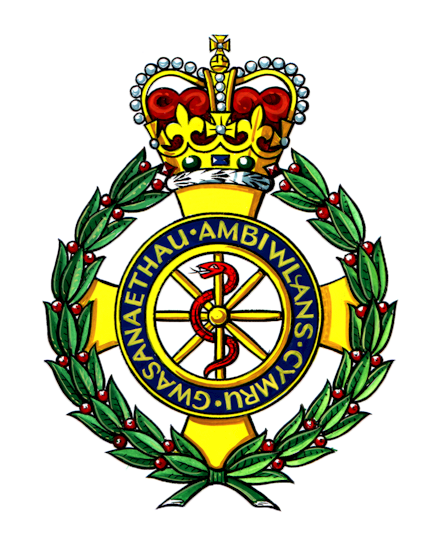


***Ymddiriedolaeth GIG Gwasanaethau Ambiwlans Cymru***

***Welsh Ambulance Services NHS Trust***

**SAFER1 Study**

**Participant Information Sheet**

*What is the study about?*

The study will help to find out what works best for patients aged 65 or older when a 999 call is made. It will help us test a computer that supports paramedics to make decisions about patient care, in particular whether the patient needs to be taken to the Accident and Emergency Department (A&E) or can be left at home with a referral to a service that is not hospital based.

*Why have I been chosen?*

We are writing to people aged 65 or over that have recently called 999 and been attended by one of the paramedics who are taking part in the study.

*What will happen if I am included?*

In about two weeks time we will send you a questionnaire which we would like you to complete and send back to us. We will be asking about your general health and your views about the care you received. We have been careful to keep the questionnaire as short as possible. If you have difficulty completing the questionnaire, we can help you to complete it by telephone or in person – please call 01792 513432 to speak to a member of the SAFER1 team to arrange this. If there is someone who is caring for you (a relative or friend), he or she can complete the questionnaire with you. If someone does help you to fill out the questionnaire then we would like to know and there is a tick box on the front page for you to fill in.

*Medical Contacts*

If you agree to be included, we would also like to track any further 999 calls, visits to A&E or hospital stays during the one-month period following this 999 call. You will not have to do anything.

*Do I have to be included?*

No, you do not have to be included. If you do not wish to be included please complete the relevant sections of the opt-out form enclosed and return it to us in the FREEPOST envelope (no stamp required). You can participate in either part of the study, or all of the study (follow up of medical records and questionnaire completion). Just let us know if you do not wish to participate in either part or both by completing the form and sending it back to us in the FREEPOST envelope – there is no need to use a stamp for this.

*Will my care be affected?*

There will be no impact on the care you receive, whether you are included or not.

*What are the benefits to being included?*

The study is helping to improve care for older people who have a health emergency. It is testing whether some patients do better if help is provided at home rather than at A&E after a 999 call. If you are included in this study we will have more chance of understanding what works better for patients.

*Who will see my information?*

All information collected about you during the course of the study will be kept strictly confidential. Any information with your name or other identifiable details will only be seen by members of the research team.

*What if there is a problem?*

We do not believe there will be any problems arising from your being included in this study. However, if there is anything you are not happy with; please contact the study Co-ordinator (details below). If you remain unhappy and wish to complain formally, please do so through the NHS complaints procedure.

*What if I change my mind?*

If you change your mind and do not wish to continue in the study, you are free to withdraw at any time. Please let the study co-ordinator know. Your care will remain unaffected.

*Who is organising and funding the research?*

The study is organised by researchers in the School of Medicine at Swansea University, in collaboration with the Welsh Ambulance Services NHS Trust and the East of England Ambulance Service NHS Trust. It is funded by the Department of Health.

*Who has reviewed the study?*

The study has been reviewed by the Research Ethics Committee for Wales.

*Contact Details*

Study Co-ordinator: Claire Williams, School of Medicine, Swansea University

Telephone: 01792 606685
