# Supplementary material for: Support and Assessment for Fall Emergency Referrals (SAFER 1): Cluster Randomised Trial of Computerised Clinical Decision Support for Paramedics
Source: PLoS One. 2014 Sep 12;9(9):e106436. doi: 10.1371/journal.pone.0106436 (PMC4162545; doi:10.1371/journal.pone.0106436)
Supplement: File S3 — SAFER 1 Patient Questionnaire at one month. (DOCX) [file pone.0106436.s004.docx]

SAFER 1 **Questionnaire**
