# Supplementary material for: Support and Assessment for Fall Emergency Referrals (SAFER 1): Cluster Randomised Trial of Computerised Clinical Decision Support for Paramedics
Source: PLoS One. 2014 Sep 12;9(9):e106436. doi: 10.1371/journal.pone.0106436 (PMC4162545; doi:10.1371/journal.pone.0106436)
Supplement: Protocol S1 — SAFER protocol 2008. (DOC) [file pone.0106436.s005.doc]

**SAFER (Support and Assessment for Fall Emergency Referrals) Trial Research Protocol**

**1. Title**

Evaluation of the costs and benefits of computerised on-scene decision support for emergency ambulance personnel to assess and plan appropriate care for older people who have fallen: a randomised controlled trial

**2. Background to the study, including both policy relevance and related research**

*Ambulance service context*: Against a background of relentlessly increasing demand for emergency care^1^, growing knowledge that a substantial proportion of 999 callers do not need care at an A&E department^2^, and recent confirmation from the Department of Health that local responses should be developed for less serious calls, ambulance services are now exploring different ways of responding to callers across the spectrum of need. This has been reinforced by both the Reforming Emergency Care ^3^ agenda and the recently launched Department of Health ‘Taking Healthcare to the Patient: Transforming NHS Ambulance Services’^4^. Some services now have a policy of ‘No-send’ in some circumstances, many are testing the provision of further assessment and advice in place of the automatic dispatch of an ambulance, and a variety of responses are being trialled in the field including single manned cars and Emergency Care Practitioners (ECPs).

*Research evidence:* Unfortunately, the clinical, cost-effectiveness and safety of these initiatives are unknown, with these developments underpinned by little research evidence. In the face of very rapid service developments, formal evaluation has been an exception rather than the rule, despite the high risks associated with this area of NHS work. The proposed study builds on previous primary and secondary research carried out by team members, including:

1) Evaluation of telephone advice using computerised decision support software in ambulance control (TAS study)^5^

2) Evaluation of Treat and Refer protocols allowing crews to make decisions to leave patients at scene (T&R study)^6^

3) Evaluation of on-scene triage of older people who have fallen to transportation/home care (Fit to be Left study)^7^

4) Profiling of older fallers in South Wales ^8^

5) Evaluation of a paramedic practitioner service for older people in Sheffield (PPOPS)^9^

These studies have focused on the development and evaluation of provision of alternative services for 999 callers who do not clinically need to attend A&E. Each study has found that a large proportion of these calls are made up of older people who fall and for whom a 999 call is made. It is known that currently up to 10% of the 999 workload is made up of older people who have fallen (approximately 50,000 per year in London), with 40% of these calls resulting in attendance but non-conveyance – despite ambulance crews having no training nor formal protocols to make decisions to leave patients at scene. For older people who have fallen, telephone assessment and advice is usually of little benefit, but results from initial trials of on-scene triage and care planning have been encouraging ^6,7,9^. Although introduction of protocols allowing paramedics and emergency medical technicians to leave patients at the scene has been found to be feasible, their impact was limited by factors related to: cultural setting; insufficient training and support for new models of care; practical issues in implementation (reluctance to refer to paper based protocols in a face-to-face assessment); and problems in working across provider boundaries (weaknesses in systems of referral)^10^. Safety concerns have been raised also – related to both current practice, and practice following the introduction of protocols. This has reinforced some concerns highlighted by several studies in the USA focusing on triage abilities of paramedics for home care^11-13^. Early results of an evaluation of paramedic practitioners targeted to 999 calls for older people suggest, however, that a significant reduction in hospital admissions can be achieved^9^, although results concerning the safety of the service are not yet available.

*Falls:* With a growing population of older people, falls and their consequences are increasingly recognised as a priority area for clinical care, service delivery development and research. The consequences of falls in the over 65s are now well known in terms of mortality, disability, loss of independence and quality of life, as well as burden and cost for the NHS ^3^. Older fallers presenting at A&E have been shown to benefit from referral for inter-disciplinary assessment, although it is not known if these results would apply to the 999 group^4^. The National Service Framework for Older People specified that those who fall and for whom a 999 call is made should be referred to a Falls Team. However, achievement of this target is impeded by a lack of skills within the ambulance service to reliably assess older people’s needs and safely make decisions to leave patients at home, as well as a lack of referral systems for the onward care of older people who have fallen.

*Information Technology and the NHS*

Within the NHS there has been increasing recognition of the major role that information technology can play in healthcare. Strategic documents have highlighted the growing need to support patients and staff in the delivery and management of services ^14-16^. This research will contribute to the agenda of ‘informing health care’ by testing an innovative application of an IT based solution to a complex service delivery setting, testing whether IT can help to deliver ‘faster, safer and more convenient patient care’^16^.

*Application of technology in the emergency care of older people who fall*

In this study, clinical decision software (CDS), based on the system that has successfully been used in previous research with ambulance and out of hours services and modified through the T&R^6^ and Fit to be Left^7^ studies, will be used on-scene through a hand-held device to support paramedics to make triage decisions for older people who have fallen, in order to decide who should be taken to A&E and who can be left at home with self-care advice and/or onward referral to a community based service. The CDS has been developed in the UK to support nurses and paramedics to assess the clinical and social needs of patients presenting with acute problems in primary or emergency care. It is already being used by some ambulance services (e.g. Lincolnshire), out of hours services (e.g. Nottingham Emergency Medical Services) and NHS walk-in services (e.g. Gateshead). The CDS guides the user through all aspects of history-taking, examination and care planning, including referral and advice giving, and can be operated on a Tablet PC, making it ideal for use by ambulance service paramedics.

Although ambulance crews are generally receptive to using new equipment, having been trained to use increasing numbers of medical devices such as defibrillators and pulse oximeters in recent years, the acceptability of an IT based system for supporting their decision making on scene is unknown. Compliance with protocols for research, documentation and even treatment has been found to be weak in several studies ^3,4,17^. Hence a participative approach will be taken with crews and managers from the outset in order to ensure that the practical details of the development and implementation of the intervention maximise the chance of uptake and success in this evaluation.

**3. Purpose of the research**

The study addresses an important area of care – of older people who fall and require emergency aid. It combines a technological innovation with a new model of service delivery across provider boundaries. Evaluation of the costs and benefits of the technology in this complex setting will provide valuable information concerning the development of more appropriate care pathways, including linking with falls prevention strategies; and potential avoidance of hospital admissions for this vulnerable patient group.

**Study aim**: To assess costs and benefits of hand-held computerised CDS technology for the on-scene assessment and care of older people who fall and call the emergency ambulance service

**Objectives:**

**Primary objective:**

To compare between intervention and control group patients at one month and six months:

1) the number of further falls for which a 999 call is made, the time to the first of any subsequent falls; and the number of A&E attendances and hospital admissions

2) quality of life of patients and carers as well as their independence and satisfaction

3) operational ‘process’ indicators: on-scene times, job cycle times – from 999 call to ‘ambulance free’ time

4) impact on resource utilisation within the NHS and costs to patients and their families

and at each participating site, to explore

- implementation issues with service providers

**Study design**

The study is designed as a mixed methods study, utilising a factorial cluster randomised controlled trial (RCT) with a qualitative component

**Description of intervention**

The intervention being evaluated is an IT innovation of CDS software on a hand-held Tablet PC for use on scene by ambulance crews to make decisions about the clinical and social care needs of patients that have called the 999 ambulance service. The innovation is part of a complex intervention with four components: training – both clinical and technology based; decision-support guidance to assess and plan care for older fallers; the hardware and software; and referral to community based care providers. The package will be evaluated as a whole, in line with the recommendations of the MRC for evaluating complex interventions to improve health^18^, as the component parts are interdependent and should not be separated out for the sole purpose of testing.

The technology will allow the traditional ambulance service Patient Report Form to be replaced with a digital record on the Tablet PC, and trained crews will be expected to use the computer to create an electronic record for every call. When the crew member attends a patient that meets the inclusion criteria for this study, the additional functionality of the decision support for assessing falls will also be used to assess whether the older person who has fallen should be taken to A&E or offered an alternative care plan. The CDS prompts the assessment and examination of any injuries associated with the fall, as well as co-morbidity that may have contributed to the fall (such as breathlessness or chest pain) and the patient’s psycho-social needs (such as their mental state and their ability to undertake activities of daily living). An assessment of environmental risk is also included. Based on these assessments, the CDS suggests an appropriate care plan (such as patient advice, referral to specific community based services etc.).

Paramedics allocated to the intervention group will receive additional training (two days) in the use of the CDS. Following initial training there will be a pre-trial period of one month during which trained crews will be expected to practise using the hand-held computer in place of paper Patient Record Forms, and the CDS for falls assessment as and when appropriate. Towards the end of this period, their use of the CDS will be audited to ensure that they have achieved proficiency. Training will be carried out with groups of 4 paramedics, and will last two days, split so that the initial training is followed up by a refresher training session/competency test after three weeks of practice. Training consists of systematic demonstration of the mechanics and functionality of the software coupled with practice and supervised structured and unstructured role play. Critical reflection and discussion is undertaken and encouraged throughout the training programme. Knowledge reviews are carried out at certain points of the process to ensure competence and understanding of key aspects of the software functionality.

To meet the primary study objective, the technology will be tested with paramedics at two study sites (S Wales, Great Western Ambulance Service). It is intended that crews that have been trained in the intervention will make decisions that can enable community-based care and avoid hospital attendance and admission for these older patients, without undertaking the lengthy, comprehensive and expensive training that is required to enable them to act as fully autonomous practitioners. Building on research findings from previous studies carried out by members of the research team, the technology will form an integral part of the package to be evaluated. Protocols have been shown to have limited impact, but integrated into on-line decision support software to be used interactively on scene, may allow identification of patients who can be safely left at home. Thus patients may benefit from a more appropriate response in their homes rather than the traditional care of transportation to A&E, or non-conveyance without referral - because they have been left at home outside protocols. The study will be powered to detect differences in processes and outcomes between patients in the intervention and control groups in these two sites.

**Sample information on size, type, location and whether the sample will be representative or not**

The study will take place in two ambulance service sites who have expressed enthusiasm concerning participation in this timely trial - South Wales and Great Western. Paramedics eligible for inclusion in the study will be randomly selected to the intervention or control groups. In the two sites, half of the recruited paramedics based at the ambulance stations in the catchment area of 1 District General Hospital with a full A&E department service will be trained to use the CDS algorithms and the hand-held device for face-to-face consultation. All older patients that have had falls and are attended by crews during the data collection period will be included in the sample. Randomisation and recruitment to the trial will be facilitated and monitored through WORTH (Wales Organisation for Randomised Trials in Health). Those not attended by an intervention group paramedic will receive the standard treatment. As it will not be practical to blind the dispatchers in ambulance control to the study group of paramedics, it will be necessary to monitor and potentially manage ambulance dispatch to avoid any selection bias, which might manifest itself in a higher pick-up or recruitment rate in the intervention group. Paramedics allocated to the intervention group will receive additional training (four days) and the hardware, as well as protocols and telephone/fax numbers for referral.

*Inclusion criteria:* 999 call for patient aged over 65 attended by a study paramedic and classified by them as having fallen. In order to maximise generalisability of findings to this group of frail patients with complex needs no patients will be excluded due to other conditions or competence. Where necessary, outcomes will be followed up with carers.

*Sample size*: The sample size for the two paramedic sites (S Wales and Great Western) is calculated for the primary objective, and with 20 paramedics (ten in each intervention group, 10 in each control group) at each site. Based on ambulance service data from the participating services, it is expected to recruit 250 older people who have fallen in each site each month. However, it may not be possible to identify all those who have fallen as eligible for inclusion in the study from information given during the 999 call, and some patients will not consent to be included. Using a conservative estimate of the recruitment of 80 older people who have fallen attended by study paramedics per month, a period of nine months will allow inclusion of 720 patients per site (1440 in total). With the 20 clusters, this will give more than 80% power at the 5% significance level to detect a drop in further 999 calls for falls from 50% (as found in London recently^6^) to 40%, assuming an intra-class correlation of less than 0.02, and to detect an effect size of 0.20 (i.e. one fifth of the population standard deviation) in SF12 scores.

**Methods to be used**

A cluster randomised trial methodology will be used, rather than random allocation of treatment at individual patient level. This has been recommended by the MRC as an appropriate approach in this circumstance, ‘where the intervention is targeted at health professionals, with the aim of studying its impact on patient outcomes’^19^. Although the use of a cluster randomisation design does raise scientific and ethical issues, it is the only practical approach where paramedics are trained to use the CDS and will not be able to ‘switch on and off’ the learned assessment skills. The issues raised will be tackled within the study through appropriate randomisation, consent procedures and data analysis techniques.

*Consent*: As emergency callers are often in distress and in need of urgent aid, consent will not be attempted by phone or at the time when the patient is first attended. Patients will be identified from routinely available ambulance service information (control room and patient record forms) and will be contacted by post at their usual home and any temporary address (such as their hospital ward) 7 – 10 days after the index fall to inform them about the study. Patients or carers will be asked to return a form if they do not wish to be contacted again or if they do not wish their medical details to be accessed for the study. Although this type of ‘opt-out’ is unusual, we have recently used the approach in two studies that have received ethical approval to include patients in this vulnerable and hard to reach group in order to improve their care. This will be confirmed with the MREC before the study.

*Outcomes:* Patients will be tracked through the 999 system, A&E departments, GPs and coroners to identify further contacts with these services (or death) following a fall within six months. Referrals will be followed up and contacts with other service providers recorded. Quality of life of patients and carers, self-reported falls and independence will be followed up by questionnaire, to be administered by post (or interview, where necessary), including the SF12^20^ , EQ5D^21^ and the CGI^22^ at 1 and 6 months; patient satisfaction will also be followed up at 1 month with the Quality of Care Monitor^23^. Despite using several validated outcome measures, care has been taken to select brief tools and efforts will be made to keep the questionnaire as short as possible in order to minimise the burden on respondents and to maximise response rate. Job cycle times will be recorded for all calls meeting the study inclusion criteria, from routine ambulance records.

*Health economics:* The economic analysis will estimate the costs of providing the new intervention, the consequences of the scheme for the wider NHS (eg A&E attendances, inpatient admissions) and the costs to patients and families. Data on the use of the health service resources will be collected for each patient using a combination of paramedic records, routine hospital records (for hospital events) and patient-completed questionnaires. Costs will then be calculated using unit-costs estimated through a micro-costing study within the trial. Direct costs to the NHS will be assessed using data logged as a part of routine practice and from resource utilisation recording sheets, together with reference to patient records and discussions with relevant finance staff. The EQ5D will also be used to estimate the quality adjusted life years (QALYs) gained from the intervention and an incremental cost-per-QALY estimated. These ratios will be presented along with their associated cost-effectiveness acceptability curves. Sensitivity analysis will be undertaken to assess the robustness of the results to changes in the configuration of the scheme and other health service costs.

*Qualitative methods*: A subset of 10 older people who fall and are attended by ambulance crews using the new technology at each study site (20 in total) will be interviewed in more depth, using a semi-structured interview schedule to ascertain their views and preferences regarding the service they received. Where possible, to minimise disruption and number of visitors, these interviews will be carried out by the community based providers who visit the older people following referral from the 999 service. The study researchers will train these providers in qualitative interviewing techniques to ensure that standards of data collection are maintained.

Semi-structured interviews will also be carried out with paramedics prior to and following their use of the cCDS technology. Focus groups (n = 6) will be carried out with stakeholders based in other service providers, particularly in the falls services to explore: triage decision-making processes; views concerning the care of older people who fall; issues in implementation of the innovation and referral.

Discussions will be taped and transcribed.

**Plans for data analysis**

Measures of process, outcome and cost will be compared between intervention and control groups patients according to ‘intention to treat’. As we expect a high rate of subsequent 999 calls for falls, it is likely that people may be attended for a fall on more than one occasion during the study period. Although, if the intervention is effective, the impact on outcomes may potentially be diluted by subsequent attendance by a paramedic or ECP using the computerised CDS, individuals will remain, for the purpose of the primary analysis, in the group to which they are first allocated. Time interval to first subsequent fall will also be compared between the two groups. Design and methods will comply with the standards laid down in the CONSORT guidelines^21^. Qualitative data will be analysed thematically using a content analysis approach.

**Scheduling of the study**

Months 1-8 modification of software and agreement of protocols for referral etc

gain ethics approval

7 – 9 paramedic recruitment, training, pilot data collection

9 – 17 patient recruitment

9 – 22 patient/carer follow up (postal questionnaires and interviews)

21 – 23 completion of follow up with patients, 999, A&E and GPs

22 – 25 focus groups with crews and stakeholders

25 – 28 analysis of data

28 - 30 interpretation and write up of findings

**Project management**

This multi centre study will be led from Swansea by Dr Helen Snooks, but will require close collaboration with research partners in England and Wales, as well as with our provider partners in S Wales and Great Western. Professor Jeremy Dale will oversee the work of the researcher to be based in Warwick, although overall project management will remain with the Swansea team, to be co-ordinated by B Wells on behalf of the project lead. The research team has extensive experience of working across academic and provider sites in previous studies in the pre-hospital setting, with timely delivery. Several of the co-applicants have previously collaborated with each other successfully in multi-centre studies across ambulance service sites, and have built strong working relationships with each other and with clinical and operational partners.

The full Project Steering Group (with all co-applicants and provider representatives) will meet quarterly, with local meetings of project implementation team (researchers, local leads and provider organisations) to manage project implementation and agree local referral pathways, to be held on a monthly basis. Allocation of responsibilities will be clear and transparent, with co-applicant responsibilities detailed in section 4, and will be confirmed at the outset of the study. The researchers will then ensure that commitments are fulfilled and project timescales adhered to.

To ensure that user involvement in the study is as high as possible, we propose to hold 2 user groups to advise on practical detail of study design, implementation and interpretation of study findings, to meet at start, middle and towards end of project:

1. with representatives of older people (e.g. Age Concern, Care Home personnel)
2. with ambulance service (managers and paramedics) and falls/primary care team (nurses/social workers etc)

**Strengths of the team**

The research team includes leading academics in the fields of: pre-hospital and emergency care research; IT implementation and human/technology interface; care of the elderly, specifically falls and injury research; primary care; epidemiology; social care research; health economics and trials methodology. The team has a strong track record in completing and publishing research findings which have contributed to the shaping of national policy and informed local clinical and operational service development.

**Likely study outputs (content and form), Methods for disseminating and implementing research**

This research will be completed at a time when ambulance services and others concerned with the care of older people who have fallen are keen to implement new models of care, and research evidence concerning the safety, effectiveness and costs of this innovation will be welcomed. The research team have a track record of publishing results from studies in prehospital emergency care, and several members have close links with services at a strategic and operational level. Findings will be disseminated directly to ambulance services, and to central IT policy leads, as well as through academic peer-reviewed journals to ensure widest readership.

**References**

1. Department of Health Government Statistical Service. Statistical Bulletin. Ambulance Services, England: 1998-9. Bulletin 1999/16, Published June 1999. ISBN 184182 053 9
2. Snooks H, Williams S, Crouch R, Foster T, Hartley-Sharpe C, Dale J. NHS emergency response to 999 calls: alternatives for cases that are neither life-threatening nor serious. *BMJ*. 2002; 325:330-333
3. National Service Framework for Older People. Department of Health 2001
4. Close J, Ellis M, Hooper R, Glucksman E, Jackson S, Swift C. Prevention of Falls in the Elderly Trial (PROFET); a randomised control trial. *Lancet*, 1999: 353 (9147): 93-97
5. Dale J, Williams S, Foster T, Higgins J, Snooks H, Crouch R, Hartley-Sharpe C, Glucksman E. Safety of telephone consultation for ‘non-serious’ emergency ambulance service patients. *Quality and Safety in Health Care*, 2004, in press
6. Snooks H, Kearsley N, Dale J, Halter M, Redhead J, Cheung WY. Towards primary care for non-serious callers to the emergency ambulance service: results of a controlled study of ‘Treat and Refer’ protocols for ambulance crews. *Quality and Safety in Health Care*, 2004; 13: 435-443
7. Fit to be left: can ambulance staff use an assessment tool to decide if an older person who has fallen can be safely left at home? Department of Health Research Programme to Support Implementation of the National Service Framework for Older People - due to report January 2005
8. Snooks HA, Close J, Gaze S, Halter M, Lyons R, Lervy B, McCabe M, Watkins A, Woollard M, Wani M, Youren A. Older fallers attended by the ambulance service and left at home: risks and opportunities, Final Report to WORD, February 2003
9. Mason S, Wardrope J, Perrin J. Developing a community paramedic practitioner intermediate care support scheme for older people with minor conditions. Emerg Med J 2003;20:196-8
10. Snooks H, Kearsley N, Dale J, Halter M, Redhead J, Foster J. Gaps between policy, protocols and practice: a qualitative study of the views and practice of emergency ambulance staff concerning the care of patients with non-urgent needs. Qual Saf Health care 2005; in press
11. Sasser SM, Brokaw M, Blackwell TH. Paramedics vs emergency physician decisions regarding the need for emergency department evaluation. (Abstract) Acad Emerg Med 1998;5:391
12. Hauswald M. Can paramedics safely decide who does not need an ambulance transport or emergency department care? (Abstract). Acad Emerg Med 1998;5:390
13. Evaluation of protocols allowing emergency medical technicians to determine the need for treatment and transport. Acad Emerg Med 2000;7:663-9
14. NHS Executive. Information for Health: an information strategy for the modern NHS 1998-2005 (September 1998)
15. Department of Health. Delivering 21^st^ Century support to the NHS: National Strategic Plan. 2002
16. NHS Connecting for Health. National Programme for IT (NPfIT). http://www.connectingforhealth.nhs.uk
17. Snooks H, Foster T, Nicholl J. Results of an evaluation of the effectiveness of triage and direct transportation to minor injuries units by ambulance crews. Emerg Med J 2004;21:105-111
18. Medical Research Council. A Framework for development and evaluation of RCTs for complex interventions to improve health. April 2000
19. Cluster Randomised trials: Methodological and ethical considerations. MRC clinical trials series. Medical Research Council November 2002
20. Ware J, Kosinski M, Keller S. A 1Item Short-form Health Survey: Construction of scales and preliminary tests of reliability and validity. Medical Care. 1996;34:220-233
21. The Euroqol Group. EuroQol – a new facility for the measurement of health related quality of life. Health Policy, 199016:199-208Drummond M, O’Brien B, Stoddart G, Torrance G. Methods for the economic evaluation of health care programmes, 2^nd^ edition. Oxford: Oxford University Press, 1997
22. Williams JG, Bayer AJ, Cheung WY et al.  Quantifying the effects of caring on carers' quality of life.  Report to WORD.  July 2002
23. Carey RC, Seibert JH. A patient survey system to measure quality improvement questionnaire reliability and validity. Medical Care, 1993;31(9):834-45
24. Moher D, Schulz KF, Altman D for the CONSORT group. The CONSORT statement: revised recommendations for improving the quality of reports of parallel group randomised trials. JAMA. 2001;285:1987-1991
